# Phoretic interactions and oscillations in active suspensions of growing *Escherichia coli*

**DOI:** 10.1098/rsos.180008

**Published:** 2018-05-30

**Authors:** Remigijus Šimkus, Rita Meškienė, Agota Aučynaitė, Žilvinas Ledas, Romas Baronas, Rolandas Meškys

**Affiliations:** 1 Institute of Biochemistry, Life Sciences Center, Vilnius University, Sauletekio al. 7, 10257 Vilnius, Lithuania; 2 Faculty of Mathematics and Informatics, Vilnius University, Naugarduko 24, 03225 Vilnius, Lithuania

**Keywords:** bacterial growth, bacterial chemotaxis, biochemical oscillations, Janus particles, self-phoresis

## Abstract

Bioluminescence imaging experiments were carried out to characterize spatio-temporal patterns of bacterial self-organization in active suspensions (cultures) of bioluminescent *Escherichia coli* and its mutants. An analysis of the effects of mutations shows that spatio-temporal patterns formed in standard microtitre plates are not related to the chemotaxis system of bacteria. In fact, these patterns are strongly dependent on the properties of mutants that characterize them as self-phoretic (non-flagellar) swimmers. In particular, the observed patterns are essentially dependent on the efficiency of proton translocation across membranes and the smoothness of the cell surface. These characteristics can be associated, respectively, with the surface activity and the phoretic mobility of a colloidal swimmer. An analysis of the experimental data together with mathematical modelling of pattern formation suggests the following: (1) pattern-forming processes can be described by Keller–Segel-type models of chemotaxis with logistic cell kinetics; (2) active cells can be seen as biochemical oscillators that exhibit phoretic drift and alignment; and (3) the spatio-temporal patterns in a suspension of growing *E. coli* form due to phoretic interactions between oscillating cells of high metabolic activity.

## 1. Introduction

Active colloids are particles that self-propel through viscous fluids by converting energy extracted from their environment into directed motion [1–4]. Perhaps the most prominent examples of artificial and natural active colloids are chemical reaction-driven Janus particles and living bacteria, respectively [1–4]. It is thought that these types of active particles differ by their mechanisms of self-propulsion and chemotaxis. In the case of *Escherichia coli* and many other bacteria, the chemosensory and chemotaxis signal transduction systems enable the movement of cells towards attractants and away from repellents by controlling the rotational direction of their flagellar motors [5]. Chemotactic *E. coli* are capable of forming sophisticated patterns of self-organization on agar plates [6,7]. In turn, artificial Janus swimmers [8,9] are transported due to phoresis [4], which is defined as any sort of colloidal migration in gradients of all kinds [10]: solute concentration, electrical field, temperature and so on. The behaviour of self-phoretic micro-swimmers may resemble the behaviour of a living cell by exhibiting ‘colloidal' chemotaxis along the self-generated chemical gradients [11–16] or gradients of fuel [17,18]. It is thought that synthetic chemotactic colloids exhibit chemotactic behaviour due to interplay between self-propulsion, phoretic drift and alignment, driven by and mediated via chemicals [12]. Moreover, the chemotaxis of synthetic colloids is sometimes modelled by chemotaxis–diffusion equations (Keller–Segel model) which are normally used to model the chemotactic behaviour of living cells [2,11–16,19]. Recently, a representative model for phoretically interacting active colloids was introduced [19]. It was shown, in particular, that phoretic interactions induce pattern formation in typical Janus colloids when the lifetime of the phoretic field is not too small compared to the persistence time of a phoretic swimmer. Although phoretic effects have been the focus of many theoretical and experimental studies, there is a lack of insights into the role of such effects in suspensions of living cells. The complexity of transport of *E. coli* in liquid suspensions was revealed recently by Schwarz-Linek *et al.* [3].

The main goal of this paper is to understand the role of phoretic interactions in self-organization of bacteria. More specifically, we investigate whether phoretic interactions are able to enforce the formation of spatio-temporal patterns in liquid suspensions of bacteria.

It has been recently shown that non-invasive bioluminescence imaging can be applied to provide new insights into bacterial migration and self-organization in liquid cultures [20–24]. On the basis of the analysis of imaging data, it was hypothesized in [21,23,24] that the phoretic transport and pH-taxis of metabolically active aerobic cells may be responsible for the formation of the oscillating (or ‘merging–emerging') patterns of chemotaxis (for the corresponding mathematical models, see [25–29]). This study is designed to elucidate the possible role of phoretic interactions in self-organization of bacteria. The paper makes the following contributions:

— A number of long-run (24 h) experiments to characterize self-organization of *E. coli* and its mutants in standard microtitre plates are described. Mutants that potentially differ with respect to their ability to swim were chosen for the investigation.
— The results of mathematical modelling of pattern formation are presented. The model is based on Keller–Segel equations of chemotaxis with logistic cell kinetics.
— The underlying mechanisms of bacterial self-organization in liquid cultures of *E. coli* are proposed. It is argued that patterns are formed due to phoretic interactions between cells that exhibit biochemical oscillations [30,31].

## 2. Material and methods

### 2.1. Cultures and mutants

The cultures of *E. coli* and their mutants were prepared as described in [20,21,23,24]. Cells were grown in LB (Luria–Bertani) broth medium. To start the growth, 1 ml of overnight culture stock was added to 100 ml of LB medium. The cells were aerobically cultured in Erlenmeyer flasks on a rotary shaker at 30°C. Optical density measurements at 600 nm (OD600) were used to monitor the concentration of cells. The freshly harvested mid-log phase cultures (OD600 = 0.4–0.6) were chosen for examination [20,21,23,24]. The induction of the culture was provided by adding a stock solution of isopropyl-β-d-thiogalactoside into LB medium up to a final concentration of 1 mM.

The Quick and Easy *E. coli* Gene Deletion Kit (Gene Bridges) was used for generation of deletion mutants. Functional cassettes for ndh, nuoA, cydA, cyoA genes flanked by homology arms were prepared by polymerase chain reaction (PCR) (50 µl): 10× Taq buffer (5 µl), 2 mM dNTP (8.75 µl), 10 µM primer Fw (3.2 µl), 10 µM primer Rv (3.2 µl), 25 mM MgCl_2_ (5 µl), FRT-PGK-gb2-neo-FRT PCR-template (1 µl), 5 U µl^−1^ Taq polymerase (0.5 µl) and H_2_O (23.35 µl); one cycle (95°C: 3 min), 10 cycles (95°C: 30 s; 57°C: 30 s; 68°C: 2 min 40 s), 20 cycles (95°C: 30 s; 57°C: 30 s; 68°C: 2 min 40 s + 20 s every cycle). The PCR products were purified by running the whole PCR sample on an agarose gel and subsequent gel extraction using the GeneJET PCR Purification Kit (Thermo Fisher Scientific). DNA concentration was adjusted to 100–400 ng µl^−1^.

Transformation of *E. coli* DH10*β* (F- *end*A1 *rec*A1 *gal*E15 *gal*K16 n*up*G *rp*sL Δ*lac*X74 *Φ*80*lac*Z*Δ*M15 *ara*D139 Δ(*ara*,*leu*)7697 *mcr*A *Δ*(*mrr*-*hsd*RMS-*mcr*BC) λ-) with Red/ET expression plasmid pRedET (mediating ampicillin resistance) and disruption of a chromosomal DNA fragment by the FRT-flanked PGK-gb2-neo cassette (mediating kanamycin resistance) were performed as described in the technical protocol of Quick and Easy E. coli Gene Deletion Kit, v. 2.3. Verification of the successfully modified genome by PCR analysis was performed using oligonucleotides primer 2, primer 3, 10BndhtkrUS, 10BndhtkrDS, 10BnuoAtkrUS, 10BnuoAtkrDS, 10BtkrcydAUS, 10BtkrcydADS, 10BcyoAtkrUS and 10BcyoAtkrDS and Maxima Hot Start PCR Master Mix (Thermo Fisher Scientific), according to the manufacturer's recommendations. PCR primer2 and primer3 is designed to verify the correct insertion of the FRT-flanked kanamycin/neomycin resistance marker cassette, and the other primers are designed to verify whether the gene of interest is undisrupted, disrupted or replaced with the FRT-flanked cassette.

The oligonucleotides below were used to add the 50 bp homology regions for Red/ET recombination to the FRT-PGK-gb2-neo-FRT cassette:

10Bndh::kanFw: tgttaataaccattaattaacaattggttaataaatttaagggggtcacgaattaaccctcactaaagggcg; 10Bndh::kanRv: tcctcaagggcggatgaggcgtttatgccacatccgccagtgtacgtcgataatacgactcactatagggctc; 10BcydA::kanFw: tctaacggggttcactctcggagtcttcatgcgatgagcaaggagtcatgaattaaccctcactaaagggcg; 10BcydA::kanRv: caccagataaaacgcaatacttcataatcgatcatttgacgactcctgtctaatacgactcactatagggctc; 10BcyoA::kanFw: tgcccacacactttaaacgccaccagatcccgtggaattgaggtcgttaaaattaaccctcactaaagggcg; 10BcyoA::kanRv: actgcatcaagtgataattttccgaacatctttattcttcctcaaccccttaatacgactcactatagggctc.

The oligonucleotides below were used for verification of the successfully modified genome by PCR analysis: primer2: cgagactagtgagacgtgctac; primer3: tatcaggacatagcgttggctacc; 10BndhtkrUS: cgctcaaataataaacaataaactctgt; 10BndhtkrDS: ccaacattgatttattcacgggaa; 10BnuoAtkrUS: ttccattgcttcacaacgga; 10BnuoAtkrDS: gcatctggtcatacagacgc; 10BtkrcydAUS: tgcaaatttgcttcaacaaaaacc; 10BtkrcydADS: agtgtggtgcaatggagtta; 10BcyoAtkrUS: ttctcatcacccagttgtcac; 10BcyoAtkrDS: caatcgccacgatgatatacatg.

Other *E. coli* mutants were from the Keio collection [32]. All mutants were transformed by pSB417 plasmid, which harbours the *luxCDABE* genes from *Photorhabdus luminescens* placed under the control of the lac promoter [33].

### 2.2. Image data analysis and numerical methods

Microtitre plate-based assays were performed. Black flat bottom 96-well microtitre plates were used (Greiner Bio-one GmbH, Germany). In each microtitre plate test experiment the bioluminescence images of 12 adjacent wells (three rows, four columns) filled with 0.25 ml of liquid cultures of control and various mutant cells were acquired simultaneously using a CCD camera CoolSNAPcf (Photometrics, USA) equipped with a Schneider Kreuznach Xenon XN 0.95/25 objective. The experiments were carried out in a light-tight box at 22°C. The frames were taken once per minute for 24 h with an exposure of 1 min. The bioluminescence imaging data were collected using the image processing software Image-Pro Express V. 6.3. The freshly harvested cultures of each mutant were tested three times in 2–4 wells. Bioluminescence images (1440 images per sample) of 7–11 samples of each mutant were analysed.

Software with functions to extract pseudo-one-dimensional spatio-temporal plots from the two-dimensional images and to isolate and count high-concentration area aggregates was developed. Quantification of the self-organization process by counting the average number of unstable aggregates was performed using this software. Signal processing techniques were used to determine such an average number of aggregates. First, the bioluminescence images were pre-processed for noise reduction by a one-dimensional Gaussian filter in *x*-direction (each horizontal line) [34]. Second, the Otsu method [35] was used to determine a threshold for each line. With the applied threshold, the number of highlighted areas at each time step was calculated. Lazarus integrated development environment [36] and Free Pascal [37] were used to develop this software.

Owing to the nonlinearity of governing equations of the Keller–Segel–Fisher model [21–24] used, an exact analytical solution does not exist for the initial boundary value problem. Hence, the bacterial self-organization was simulated numerically using the finite-difference technique [38]. A uniform discrete grid was used for the space (*x*) dimension and for the time. An explicit finite-difference scheme has been built as a result of the difference approximation of the equations [38]. Verification of the scheme has been performed through varying step sizes and error tolerances. In the simulation, there were 224 grid points on the *x*-axis. Also a constant dimensionless step size of 0.00005 was used in the time direction. Very similar mesh sizes of space and time were used by Ei *et al.* [27] for simulating spatio-temporal oscillations in the Keller–Segel system. A software tool implementing computational schemes was developed using Free Pascal [37].

## 3. Results and discussion

### 3.1. Patterns of bioluminescence

**Figure 1.**
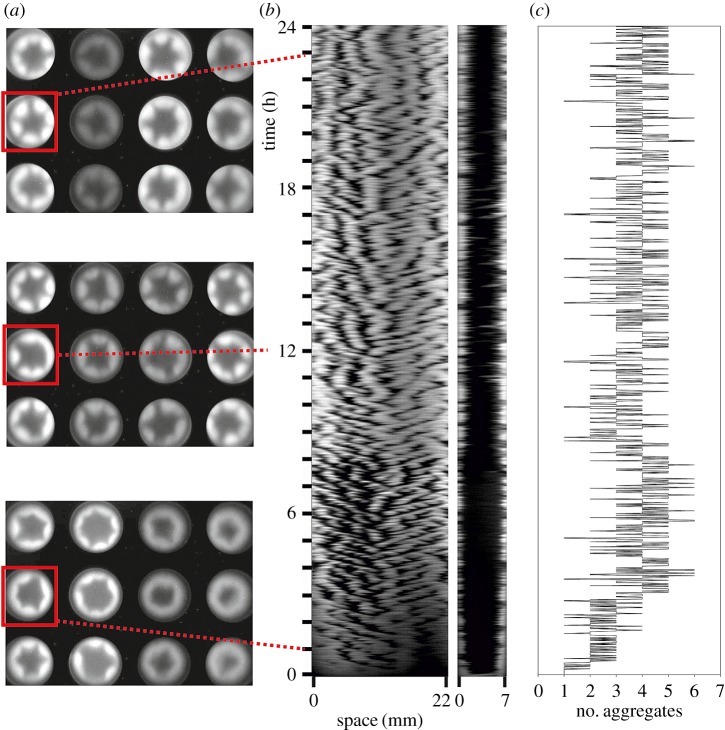
Spatio-temporal patterns of bacterial luminescence. (*a*) Examples of images taken at different times. Images of suspensions of *E. coli* (first column from the left) and its mutants deficient in NhaA (second column), NhaB (third column), ChaA (fourth column) were taken 1, 12 and 23 h after plate filling. The ratio between luminescence intensities in the brightest and the darkest areas in each sample is approximately 2.5. (*b*) Example space–time plots (kymographs) obtained from the data of bioluminescence intensity measurements along the three-phase contact line (22 mm) and across the diameter (7 mm) of the microtitre plate well. The corresponding well is marked by the square in (*a*). (*c*) The results of the corresponding (*b*) data processing are represented by the number of aggregates fluctuating over time.

Patterns of inhomogeneous bioluminescence were observed in microtitre plate wells filled with cell suspensions in growth medium. *Escherichia coli* and its mutants show similar patterns during 24 h recordings. Illustrative snapshots are shown in figure 1*a* (see also electronic supplementary material, movie S1). The bioluminescence along the circular contact line and across the diameter of the well was measured using the image processing software [24]. Typical examples of the space–time plots of bioluminescence are shown in figure 1*b*. The patterns of bioluminescence observed during the long-run experiments can be characterized by the formation of luminous aggregates, which were also considered as azimuthal waves or plumes/clouds [20–24]. The number of aggregates in the microtitre plate well varies between one and six. The observed fluctuations in the number of aggregates during 24 h recordings are illustrated by figure 1*c*. The self-organization of bacteria in a microtitre plate well was characterized by the average number of bioluminescent aggregates (*m*) formed along the three-phase contact line [24].

### 3.2. Analysis of mutants regarding their ability to self-organize

**Figure 2.**
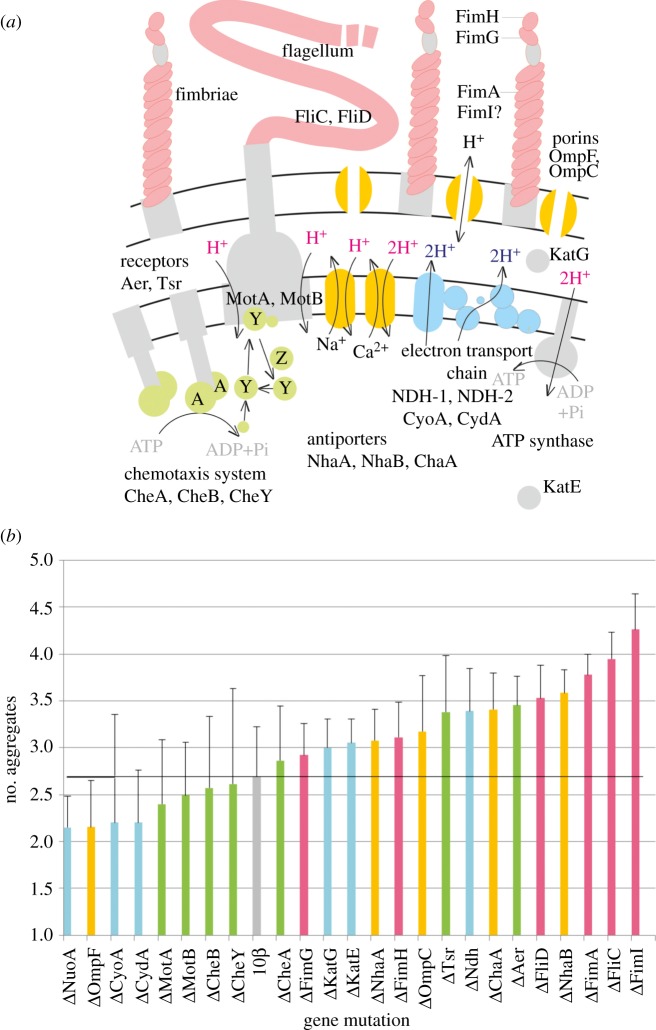
Mutations and their influence on patterns of self-organization. (*a*) A scheme of bacterial cell components, which were changed by mutations. The transport of protons across outer and inner membranes of *E. coli* is illustrated on the same figure. In each mutant, the gene encoding an individual protein was deleted. These genes are responsible for the synthesis of proteins which are related to respiration (blue), ion channels (orange), surface appendages (red) and chemotaxis (green). (*b*) The average numbers of aggregates formed by *E. coli* DH10*β* and its mutants in microtitre plate wells.

Mutants with potentially modified chemotactic or phoretic properties were chosen for our study. These mutants lack the genes encoding various structural, regulatory or transport proteins, and enzymes, which are schematically depicted in figure 2*a*. The scheme was designed in accordance with the relevant literature [39–44]. The results of the analysis of mutants for their ability to form spatio-temporal patterns are summarized in figure 2*b*. The dynamic range of changes in the average number of aggregates (*m*) is rather narrow. The ratio between the largest and the smallest values of *m* is approximately 2. Nevertheless, a semi-quantitative comparison of the effects of mutations on the patterns of self-organization can be provided.

As can be seen in figure 2*b*, pattern formation is suppressed, i.e. a small average number of luminous aggregates are formed, in suspensions of mutants deficient in the proton-translocating (two protons per one electron) dehydrogenase (NDH-1) or cytochromes (CyoA, CydA) of the electron transport chain of bacteria [39]. A similar negative effect is characteristic of bacteria deficient in a major porin (OmpF) responsible for aqueous channels in the outer membrane [40]. Contrarily, mutants deficient in non-proton-translocating dehydrogenase (NDH-2) [39], flagella components (FliC or FliD) [5], the main body of fimbriae (FimA or FimI) [41], aerotaxis receptors (Aer or Tsr) [39] or proton antiporters (NhaA, NhaB or ChaA) [42] show a slightly promoted pattern formation, i.e. a higher number of luminous aggregates are formed when compared with control bacteria. The cells deficient in chemotaxis proteins (CheA, CheB or CheY) [5] flagellar motor components (MotA or MotB) [5], catalases (KatE or KatG) [43] or adhesive tips of pili (FimH or FimG) [41] generate patterns that are closest to control. Thus, the formation of bioluminescence patterns is related neither to the chemotaxis system of bacteria nor to the rotation of flagella. Seemingly, there is an alternative mechanism of the chemotactic pattern formation in suspensions of bacteria near the three-phase contact line. We explain this mechanism as ‘colloidal' chemotaxis (i.e. phoretic drift and alignment of chemotactic colloids [12]), which is in line with recent studies of active suspensions of synthetic particles [11–19]. As follows from the analysis of the histogram shown in figure 2*b*, the average number of aggregates formed due to ‘colloidal' chemotaxis is determined by two key factors. Firstly, it depends on the efficiency of proton translocation across membranes. The efficiency is higher (and more aggregates are formed) when the stoichiometry (H^+^/e^−^ ratio) of proton translocation by the electron transport chain is higher [39] (compare the effects of dehydrogenases). The efficiency of translocation can be suppressed to some extent by hydrogen ion antiporters: mutants lacking antiporters (NhaA, NhaB, ChaA) form more aggregates than control. Secondly, the average number of chemotactic aggregates depends on the smoothness of the cell surface. ‘Bald' mutants, devoid of surface appendages (fimbriae or flagella), form a higher number of chemotactic aggregates when compared with control or other mutants, which are more ‘hairy'. For example, bald mutants deficient in fimbriae (Δ*fimI*) [41] tend to form more aggregates than hairy cells which lack just adhesive tips of fimbriae (Δ*fimG* or Δ*fimH*). It was shown recently that surface appendages strongly impact nanomechanical and electrokinetic properties of *E. coli* cells [44]. Therefore, it can be suggested that these properties may be important also in the formation of spatio-temporal patterns. In the following, we present the results of simulations of pattern-forming processes in the cultures of *E. coli* and discuss these processes assuming that active cells may behave as chemotactic colloids.

### 3.3. Mathematical modelling

#### 3.3.1. Pattern formation in terms of the Keller–Segel–Fisher model

The Keller–Segel equations can be used to model pattern formation in bacterial populations [25]. However, similar equations can also be derived for chemotactic colloids [2,11–16,19]. Below, our mathematical model of pattern-forming processes is provided, keeping in mind that active cells may behave as chemotactic colloids that exhibit phoretic interactions [19].

The dynamics of the *E. coli* population near the three-phase contact line has been described by a system of two equations, which in the dimensionless form read [21,22]

3.1 $$\begin{array}{rl} \frac{\partial n}{\partial t} & =D\Delta n-\chi \nabla (n\nabla c)+\alpha n(1-n)\\ \;\text{and}\;\frac{\partial c}{\partial t} & =\Delta c+\frac{\gamma n}{1+\beta n}-\delta c,\;x\in (0,l),\;0<t\leq T, \end{array}\}$$

where *x* and *t* stand for space and time, respectively, *n*(*x, t*) is the dimensionless cell density, *c*(*x, t*) is the dimensionless concentration of the chemoattractant (chemical signal), *D* is the diffusion coefficient, *χ* is the chemotactic sensitivity, *α* stands for the growth rate of a cell population, *β* stands for the saturating signal production, *l* is the dimensionless length of the contact line, *T* is the final time and *Δ* is the Laplace operator. The dimensionless parameters *γ* and *δ* of the production and the degradation of the chemoattractant are usually assumed to be equal to 1 [25,27], but sometimes these parameters are kept in the dimensionless model to study the spatial pattern formation [26]. All the parameters (*D*, *χ*, *α, β*, *γ*, *δ*, *l* and *T*) of the model are dimensionless and assumed to be constant. According to the classification of chemotaxis models [25], these governing equations are a combination of the saturating signal production (M6) and the cell kinetics (M8) models. Governing equations (3.1) are also known as the Murray–Myerscough system [45]. The system (3.1) with zero-flux or periodic boundary conditions generates spatio-temporal oscillations [27,29,46].

At the following values of the parameters, the system (3.1) with the periodic boundary conditions produces irregular spatio-temporal oscillations qualitatively similar to the experimentally observed structures:

3.2 D=0.1,β=0.73,χ=6,α=1,γ=δ=1,l=2πr,r=2.5,T=400,

where *r* is dimensionless radius of the circle corresponding to the contact line.

**Figure 3.**
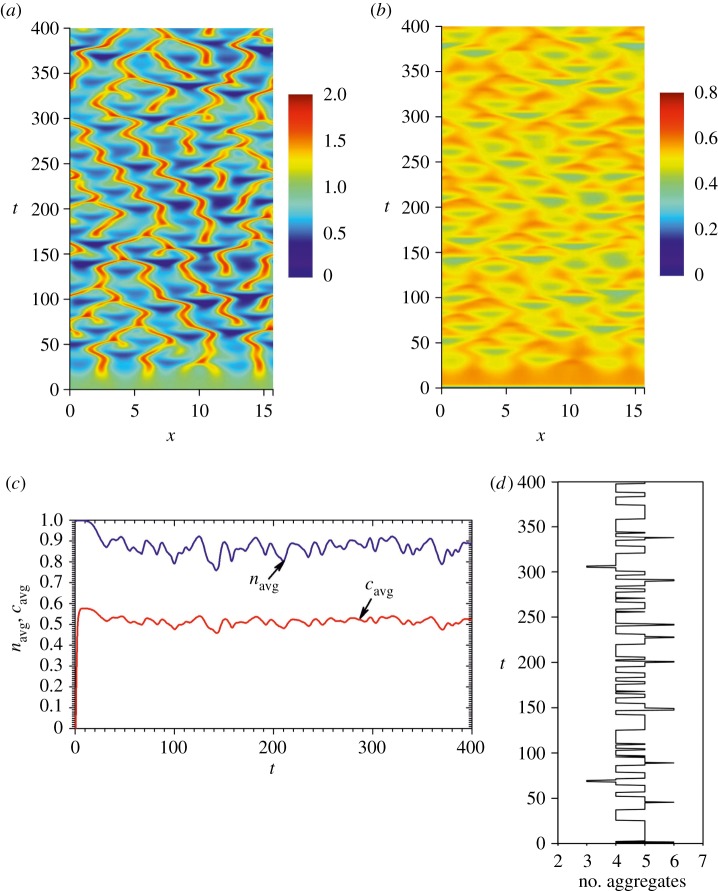
Simulated space–time plots of the dimensionless cell density *n*(*x*, *t*) (*a*) and the chemoattractant concentration *c*(*x*, *t*) (*b*), and the dynamics of the corresponding averaging values *n*_avg_(*t*) and *c*_avg_(*t*) for the contact line (*c*). The results of the corresponding (*a*) data processing are represented by the number of aggregates fluctuating over time (*d*). Values of other model parameters are as defined in equation (3.2).

Figure 3 shows numerically simulated space–time plots of the dimensionless cell density *n*(*x*,*t*), the chemoattractant concentration *c*(*x*,*t*) and the corresponding values *n*_avg_(*t*) and *c*_avg_(*t*) averaged on the contact line,

3.3 $$\begin{array}{rl} n_{\mathrm{avg}}(t) & =\frac{1}{l}\int_{0}^{l}n(x,t)\text{d}x\\ \;\text{and}\;c_{\mathrm{avg}}(t) & =\frac{1}{l}\int_{0}^{l}c(x,t)\text{d}x. \end{array}\}$$

The pattern simulation started at a 10% random initial perturbation of cells and a uniform zero concentration of chemoattractant in the medium [21,23].

As one can see in figure 3, values of *n*_avg_ and *c*_avg_ rather quickly (at *t* ≈ 10) reach the homogeneous steady-state solution (1, (*γ*/*δ*)/(1 + *β*)) ≈ (1, 0.58) of the system (3.1), while later *n*_avg_ and *c*_avg_ range notably below (0.87, 0.51), the homogeneous steady-state solution. The same effect was also noted at other values of the model parameters [22]. Additional numerical experiments assuming *γ *= *δ* = 1 showed that increasing *α*, *D*, *β* as well as decreasing *χ* leads to increasing *n*_avg_ and *c*_avg_. This feature can be also revealed by a simple analysis of the mathematical model.

#### 3.3.2. Pattern formation parameters

The necessary condition for instability of the homogeneous steady state was obtained by applying a linear stability analysis for equation (3.1) (see appendix A for details),

3.4 $$\chi >{\left(\sqrt{D\delta }+\sqrt{\alpha }\right)}^{2}\frac{{\left(1+\beta \right)}^{2}}{\gamma }.$$

This instability condition was then used to determine an interval of values for the number *m* of unstable aggregates,

3.5 $$m^{\left(2\right)}\in \left[r^{2}\left(\kappa -\sqrt{\kappa^{2}-\delta \alpha /D}\right),\;r^{2}\left(\kappa +\sqrt{\kappa^{2}-\delta \alpha /D}\right)\right],$$

where *κ *= (*χγ*/(1 + *β*)^2^ − *Dδ* − α)/2*D*.

Note that the instability condition (3.4) is independent of the domain size, while the number *m* of unstable aggregates directly depends on the dimensionless radius *r* of the microtitre plate.

**Figure 4.**
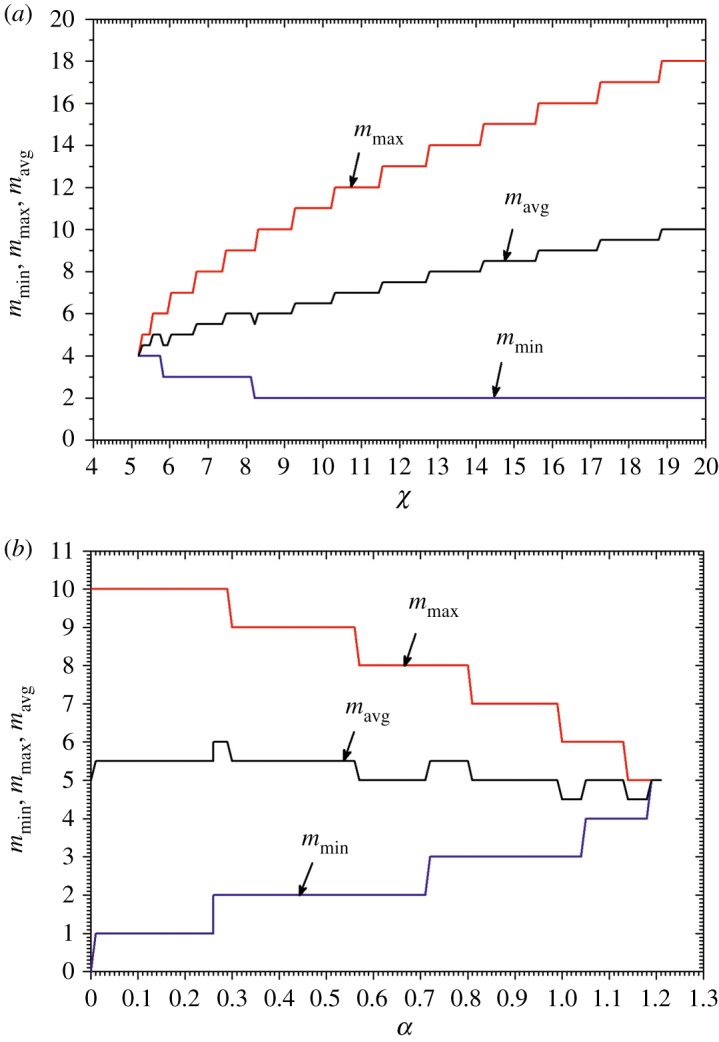
The minimal (*m*_min_), maximal (*m*_max_) and mean (*m*_avg_) numbers of unstable aggregates versus the chemotactic sensitivity *χ* at *α *= 1 (*a*) and the cell growth rate *α* at *χ* = 6 (*b*). Values of the other model parameters are as defined in equation (3.2).

Figure 4 shows the dependence of the number of unstable aggregates on the chemotactic sensitivity *χ* calculated at a certain cell growth rate *α *= 1 and on the cell growth rate *α* calculated at *χ* = 6. When calculating the numbers of aggregates, values of the other model parameters were as defined in equation (3.2).

As one can see from formulae (3.4) and (3.5), neither the instability condition nor the number of unstable aggregates depends on the cell density. As the average initial density of cells was used in defining the dimensionless parameters [22,25], the instability condition as well as the number of unstable aggregates implicitly involves also the initial density of cells. Particularly, as the dimensionless chemotactic sensitivity *χ* was defined as directly proportional to the cell density, all the effects of the *χ*-parameter can also be considered as influenced by the cell density.

The values of the dimensionless parameters of the model were determined experimentally by changing input parameters and aiming to achieve an irregular wave pattern comparable to the one shown in figure 1*b*. Taking into account the transformation of variables [22,25], one can determine the values of the dimensional parameters. Assuming that the length l* = 22 mm of the three-phase contact line and the duration T* = 4 h = 1.44 × 10^4^ s of the physical experiment to be simulated correspond to the dimensionless length (*l* = 2π*r*, where *r* = 2.5 is the plate radius) and the duration (*T* = 400) used in the pattern simulation, we calculate the dimensional diffusion coefficient of the chemoattractant (*D*_c_ = (l*/*l*)^2 ^× (*T*/T*) ≈ 5.5 × 10^−2^ mm^2^ s^−1^) and of the cells (*D*_n_ = 0.1*D*_c_ ≈ 5.5 × 10^−3^ mm^2^ s^−1^). The obtained value of the diffusion coefficient *D*_n_ of *E. coli* cells is greater than the value (2–4 × 10^–4^ mm^2^ s^−1^) determined by Berg & Turner [47]. However, it fits rather well the estimates made by Perry (2.2 × 10^–3^ mm^2^ s^−1^) [48] and by Lin *et al*. (7 × 10^–3^ mm^2^ s^−1^) [49]. The dimensional cell growth rate in our model equals *α*(*T*/T*) ≈ 0.028 s^−1^, which also means that the cell division period equals ln(2)/0.028 ≈ 25 s. This value of the cell division period is discussed below.

#### 3.3.3. Effect of the chemotactic sensitivity and the cell growth

**Figure 5.**
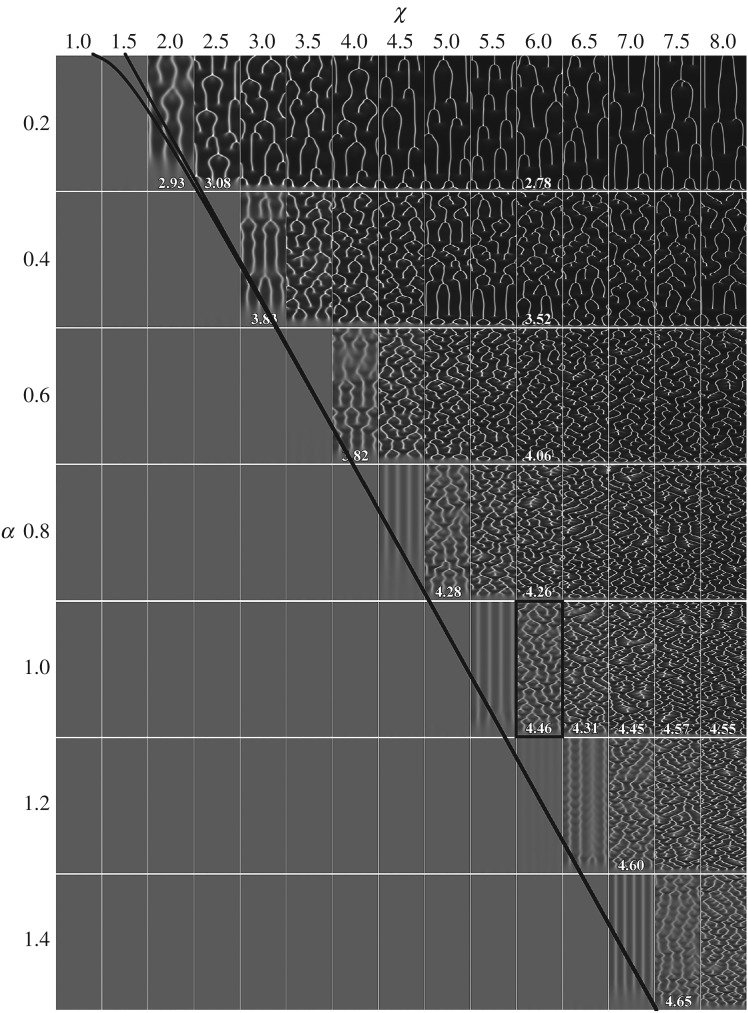
Spatio-temporal patterns of the cell density *n*(*x, t*) simulated for different values of chemotactic sensitivity *χ* and the cell growth rate *α*. The solid lines correspond to the bifurcation conditions (precise and its linear approximation) given by equation (3.6). Values of the other model parameters are as defined in equation (3.2).

Assuming *γ* = *δ *= 1 as is usual for the dimensionless Keller–Segel–Fisher model, the chemotactic sensitivity *χ* and the cell growth rate *α* are the most important parameters contributing to the occurrence of instability of the spatially homogeneous stationary solution [26,27]. Figure 5 shows spatio-temporal patterns of the cell density *n*(*x,t*) simulated for different values of the *χ*- and *α*-parameters, while values of all the other parameters are the same as defined in equation (3.2). As one can see in figure 5, the spatio-temporal oscillations appear only at relatively large values of *χ* and depend on *α*.

A dependence (3.4) of the chemotactic sensitivity *χ* on the cell growth rate *α* is depicted in figure 5 by a solid curve and its linear approximation around a value *α* = 1,

3.6 $$\chi ={\left(\sqrt{0.1}+\sqrt{\alpha }\right)}^{2}{\left(1+0.73\right)}^{2}\approx 2.99{\left(0.32+\sqrt{\alpha }\right)}^{2}\approx 3.95\alpha +1.26.$$

A similar bifurcation line in the parameter space (*χ*, *α*) was obtained when calculating hexagonal stationary solutions of a similar problem on a rectangle [27].

According to equation (3.5), the number *m* of aggregates varies from *m*_min_ = ceil(*rk*_1_) ≈ ceil(2.87) = 3 to *m*_max_ = floor(*rk*_2_) ≈ floor(6.86) = 6, where *k*_1_ ≈ 1.32 and *k*_2_ ≈ 7.53 are the unstable wavenumbers *k* (*κ* ≈ 4.52; see appendix A). The arithmetic mean of these *m*_min_ and *m*_max_ equals 4.5, which compares well with that (4.46) seen in the corresponding simulated pattern, marked by a solid rectangle in figure 5.

#### 3.3.4. Effect of the production and degradation of the chemoattractant

To investigate the influence of the production and degradation of the chemoattractant on the formation of the spatio-temporal patterns, the effect of two dimensionless parameters *γ* and *δ* in the model (3.1) was studied.

Both the parameters, *γ* and *δ*, can be eliminated from the model (3.1) by using the following rescaling:

3.7 $$x^{*}=x\sqrt{\delta },\;t^{*}=t\delta ,\;\chi^{*}=\chi \gamma /\delta ,\;\alpha^{*}=\alpha /\delta \;\text{and}\;c^{*}=c\gamma /\delta .$$

The rescaling and dropping the asterisks yields the dimensionless governing equations the same as equations (3.1) only without the parameters *γ* and *δ*, i.e. *γ* and *δ* become equal to one in equation (3.1).

According to the rescaling (3.7), a spatio-temporal pattern simulated at any values of the parameters *γ* and *δ* can be also simulated at *γ* = *δ* = 1, while values of all other parameters are transformed using (3.7). The transformation of the chemoattractant concentration *c* is irrelevant when starting with a zero concentration of the chemoattractant, *c*(*x*,0), *x* ∈ [0,*l*]. An increase in the chemoattractant production rate *γ* can be offset only by a proportional decrease in the chemotactic sensitivity *χ*. The compensation of the change on the chemoattractant degradation rate *δ* is more complicated.

**Figure 6.**
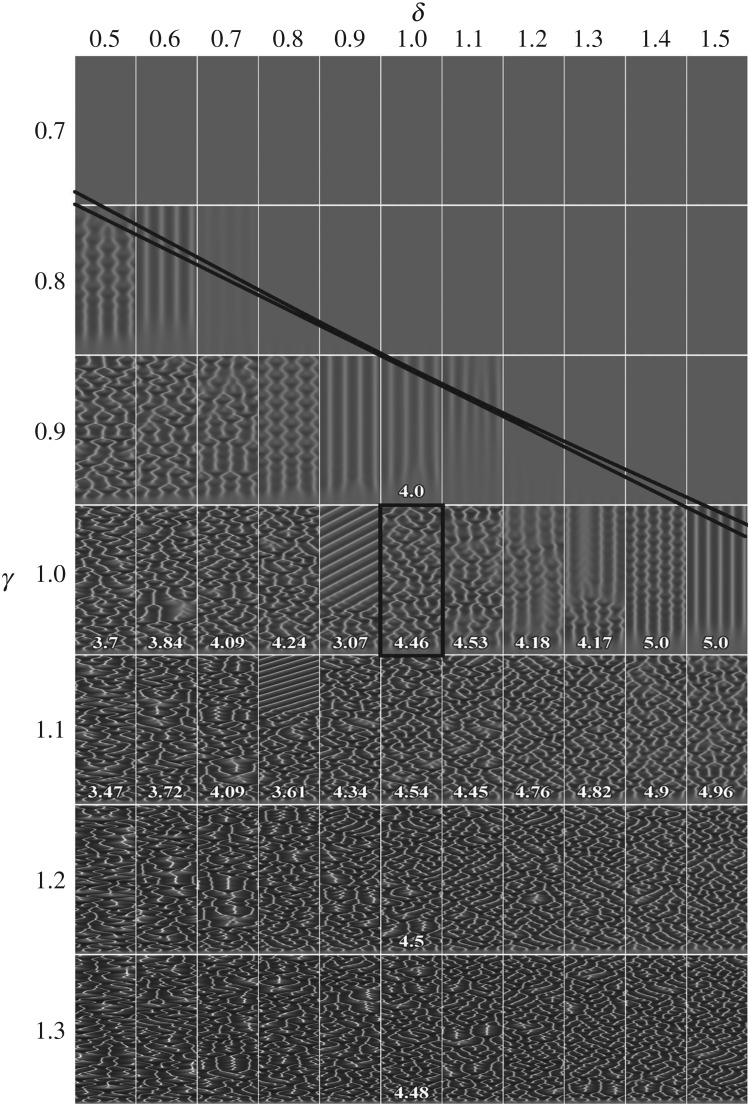
Spatio-temporal patterns of the cell density simulated for different values of the production *γ* and degradation *δ* of the chemoattractant. The solid line shows the bifurcation conditions (precise and its linear approximation) given by equation (3.8). Values of the other model parameters are as defined in equation (3.2).

To see the relationship between the production (*γ*) and degradation (*δ*) of the chemoattractant, the spatio-temporal patterns of the cell density were also simulated for different values of the *γ*- and *δ*-parameters at *α* = 1, *χ *= 6, keeping the other model parameters as defined in equation (3.2). The simulated patterns are shown in figure 6. The dependence derived from formula (3.4) as the production rate *γ* versus the degradation rate *δ* of the chemoattractant is depicted in figure 6 by a solid curve and its linear approximation around a value *δ* = 1,

3.8 $$\gamma ={\left(\sqrt{D\delta }+\sqrt{\alpha }\right)}^{2}{\left(1+\beta \right)}^{2}/\chi ={\left(\sqrt{0.1\delta }+1\right)}^{2}{\left(1+0.73\right)}^{2}/(6)\approx 0.21\delta +0.66.$$

#### 3.3.5. Relationship between chemotactic sensitivity and chemoattractant production rate

Changing only the production rate *γ* and keeping all the other model parameters unchanged results in the same spatio-temporal pattern if the chemotactic sensitivity *χ* is also changed so that the product *γχ* is kept constant.

The instability condition (3.4) can be rewritten to reveal the importance of the product *γχ* for the pattern formation,

3.9 $$\chi \gamma >{\left(\sqrt{D\delta }+\sqrt{\alpha }\right)}^{2}{\left(1+\beta \right)}^{2}.$$

According to formula (3.5), even the number of unstable aggregates directly depends on the product *γχ*.

**Figure 7.**
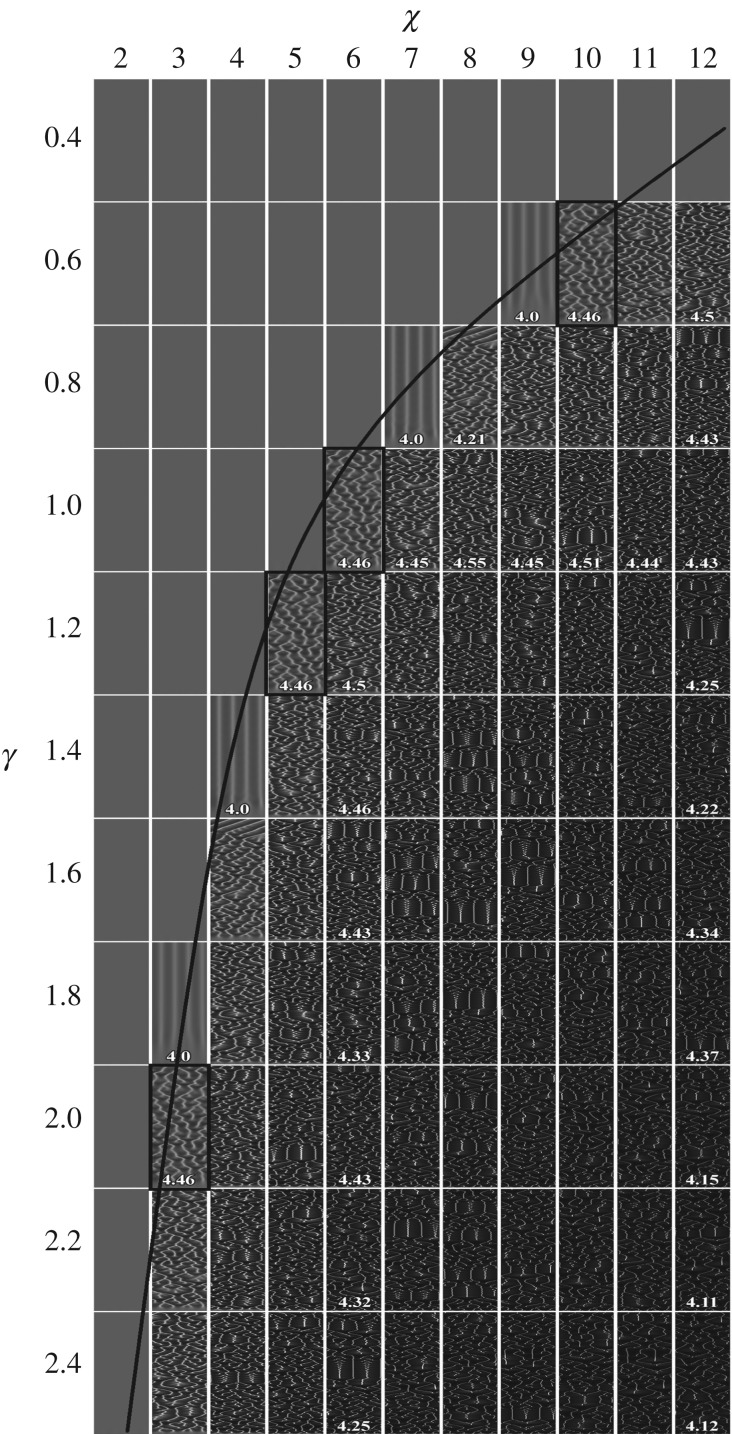
Spatio-temporal patterns of the cell density simulated for different values of chemotactic sensitivity *χ* and chemoattractant production *γ*. The solid line shows the bifurcation condition given by equation (3.10). Values of the other model parameters are as defined in equation (3.2).

As one can see in the instability conditions given by formulae (3.4) and (3.9), the chemotactic sensitivity *χ* is inversely proportional to the rate *γ* of the chemoattractant production. For a numerical study of the relationship between *χ* and *γ* as well as the influence of the parameters on the pattern formation, the patterns were simulated by changing *χ* and *γ* while all other parameters were kept constant as defined in equation (3.2) and *α *= 1. The simulated spatio-temporal patterns of the cell density are shown in figure 7. For the parameter values used in the simulations, a dependence of the chemotactic sensitivity *χ* as a function of the chemoattractant production rate *γ* can be derived from the instability condition (3.4)

3.10 $$\chi ={\left(\sqrt{0.1}+\sqrt{1}\right)}^{2}{\left(1+0.73\right)}^{2}/\gamma \approx (5.19)/\gamma .$$

This expression defines the bifurcation line *χγ* = 5.19, which is also depicted in figure 7. As one can see in this figure, the average number of unstable aggregates near the bifurcation line varies only slightly.

Four patterns marked by solid rectangles correspond to *χγ* = 6 > 5.19 and are practically identical. This sameness can be simply explained by the rescaling (3.7), which becomes very simple at *δ* = 1. As defined in formula (3.5), the number of unstable aggregates depends on the product *χγ,* which is constant on the bifurcation curve *χγ* = const. It was already shown that the average number of aggregates at *χγ* = 6 is approximately equal to 4.5. This number of aggregates compares well with those seen in the simulated patterns, close to the bifurcation line shown in figure 7.

The average number of aggregates is a monotonic increasing function of *χγ* (see appendix A). However, figure 7 shows that the average number of aggregates slightly decreases with increasing the chemotactic sensitivity *χ* as well as the rate *γ* of the chemoattractant production. So, formula (3.5) can be successfully used for estimation of the average number of aggregates only at values of the model parameters satisfying the instability condition (3.4) and being near the bifurcation line. This limitation can be explained by the linearization applied to the stability analysis resulting in formula (3.5) because the linearization is effective in predicting qualitative patterns of the system behaviour only in the neighbourhood of a stationary solution.

### 3.4. Pattern-forming processes in the growing culture

#### 3.4.1. Chemotaxis and phoretic interactions

According to model (3.1), pattern-forming bacteria grow and exhibit chemotaxis. Below we provide possible physical and biological explanations of these processes in cultures of bacteria confined in the wells of microtitre plates.

The chemotactic motion of a phoretic particle can be seen as its phoretic drift and alignment due to coupling with the ‘phoretic field' that is generated by all other colloids [19]. We consider a phoretic field is an effective chemical field whose gradient induces phoretic migration of cells. Thus, metabolically active bacteria, potentially, may move chemotactically (align and swim) like chemical reaction-driven Janus particles in the resulting self-produced gradient of *c* (phoretic field) by electrophoresis, diffusiophoresis or a similar phoretic mechanism [4,19]. The parameters *χ* and *γ* in the corresponding Keller–Segel model can be interpreted as some averages quantifying the cell surface phoretic mobility and surface activity, respectively [9,11]. Bearing in mind these remarks, it follows from formula (3.5) that the average number *m* of unstable aggregates formed in the microtitre plate well due to phoretic interactions can be evaluated,

3.11 $$m^{2}\approx r^{2}(\chi \gamma /{\left(1+\beta \right)}^{2}-D\delta -\alpha ))/2D,$$

where *r* is dimensionless radius of the circle corresponding to the three-phase contact line.

Equation (3.11) and the numerical values of model parameters imply that the number of unstable aggregates depends mainly on the product between phoretic mobility *χ* and the surface activity *γ* of phoretic cells. This result is consistent with the experimental data on the effects of mutations on spatio-temporal patterns of bacterial self-organization (figure 2 and the corresponding discussion). Indeed, it seems natural that ‘bald' mutants, devoid of surface appendages, are more mobile than control cells or other hairy mutants, and therefore they form more aggregates. Analogously, the higher H^+^/e^−^ ratio of proton translocation by the electron transport system implies a higher surface activity and higher number of unstable aggregates. Thus, the pattern-forming process in growing cultures can be associated with phoretic interactions driven and mediated by chemicals. Both the present and previous studies [21,23,24] indicate a key role of protons (hydrogen ions) in phoretic processes. It is very likely that rapidly diffusing protons are the main charged particles involved in the formation of the inhomogeneous chemical (phoretic) fields. In terms of chemotaxis, they can be considered as chemoattractants in the pH-taxis of aerobically growing and metabolically active *E. coli*.

#### 3.4.2. Growth and oscillations

**Figure 8.**
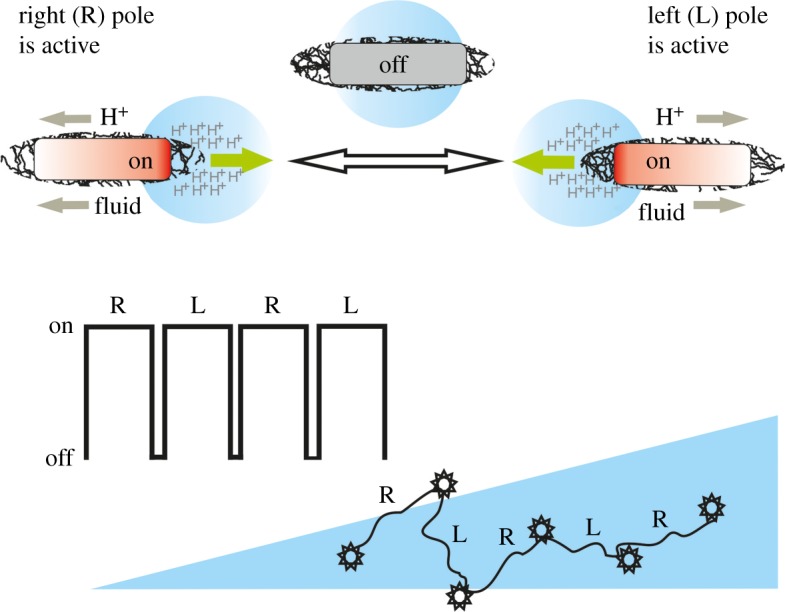
A model of aerobically growing *E. coli* cell. A metabolically active cell is considered as a pole-to-pole oscillator that moves chemotactically (curved line) like a chemical reaction-driven Janus particle in the inhomogeneous chemical (phoretic) field, which is generated by all other colloids (blue triangle). The patterns of chemotaxis were observed when the lifetime of the phoretic field is much longer than the period of oscillations.

As was shown above, the dimensional cell growth rate in our model equals 0.028 s^−1^. At first glance this result appears to be unexpected, because the rate of bacterial growth is of the order of h^−1^ [3,6,7]. In the previous studies, we interpreted similar results by assuming temporal changes of cell behaviour due to their metabolic flexibility [21,23,24]. It is known that intracellular oscillations may emerge in a living cell [30]. Despite the fact that the details of oscillatory processes, which determine the growth rate in our model, are unknown, we hypothesize that some active pole-to-pole oscillators may be involved. A well-known example of a biochemical oscillator is the Min system of *E. coli* [31]. This system positions the division plane of growing *E. coli* through pole-to-pole oscillation of Min proteins. Interestingly, the period of the swimming–resting (ON–OFF) cycle estimated above (approx. 25 s) is nearly two times shorter than the period of pole-to-pole oscillations of Min proteins in *E. coli* (approx. 50 s) [31]. In fact, this means that in both cases the bacterium becomes non-polar and passive every 25 s and we believe we are faced with the same oscillatory phenomenon (figure 8). This speculative assumption should be validated in future studies.

#### 3.4.3. Phoretic interactions between biochemical oscillators

In summary, the formation of spatio-temporal patterns in a growing culture can be explained by phoretic interactions in a population of biochemical oscillators. To characterize the relevant dynamic processes, let us return to equation (3.11). The terms in brackets in equation (3.11) represent the rates of processes in the pattern-forming diffusion–chemotaxis–growth system (3.1). We speculate that *χγ*/(1 + *β*)^2^ and *Dδ* can be interpreted, respectively, as a growth rate and degradation rate of the inhomogeneous chemical (phoretic) field. The parameter *α* can be interpreted as a growth rate of the phoretic swimmer. If so, then the system can be characterized by three temporal parameters: the duration of the phoretic field formation, *τ*_form _= (1 + *β*)^2^/*χγ*, the lifetime of the phoretic field, *τ*_phor_ = 1/*Dδ,* and the persistence time of a phoretic swimmer, *τ*_swim_ = 1/*α*. The numerical simulations indicate that the specific oscillatory patterns form when the characteristic times (as calculated from equation (3.2)) are approximately related as follows: 0.1*τ*_phor_ ∼ *τ*_swim_ ∼ 2*τ*_form_. Consequently, when *α *= 0.028 s^−1^, we have *τ*_phor_ = 360 s; *τ*_swim _= 36 s; *τ*_form_ = 18 s. These estimates indicate that phoretic interactions between short-period (approx. 0.5 min) biochemical oscillators in the growing culture of *E. coli* should be accompanied by the collective chemical oscillations (grow and decay of the phoretic field) with a period of about 6 min. These results show that the pattern-forming mechanisms in liquid medium and in agar plates [6,7] are different. Although, in both cases, the key processes are chemotaxis and growth, the origins and timescales of these processes are different.

## 4. Conclusion

The self-organization of *E. coli* cells, as detected by bioluminescence imaging, can be modelled by the Keller–Segel equations of chemotaxis with logistic cell kinetics. However, the analysis of mutants regarding their ability to self-organize shows that the observed formation of the chemotaxis patterns is related neither to chemosensory and chemotaxis signal transduction systems, nor to dynamics of flagellar motors of bacteria. This analysis together with mathematical modelling indicates that the spatio-temporal patterns in the suspensions of *E. coli* form due to phoretic interactions between oscillating cells of high metabolic activity.

## Data accessibility

The time-lapse video is provided as the electronic supplementary information accompanying this paper.

## Appendix A. Stability analysis

If the stability matrix *A_k_* has at least one eigenvalue with a positive real part, then the homogeneous steady state is unstable. For the steady state to be unstable to spatial disturbances, the determinant of the stability matrix has to be negative [25,50].

The necessary condition for instability of the homogeneous steady state can be obtained by applying a linear stability analysis for equation (3.1) around the homogeneous steady-state solution (1, (*γ*/*δ*)/(1+*β*)). The stability of the steady state depends on the temporal eigenvalues of the stability matrix of the linearized system (3.1) [26,50],

A 1 $$A_{k}=\left(\begin{array}{cc} -Dk^{2}-\alpha & \chi k^{2}\\ \gamma /{\left(1+\beta \right)}^{2} & -k^{2}-\delta \end{array}\right),$$

where *k* ≥ 0 denotes the wavenumber. On the interval *x* ∈ [0,*l*] under the periodic boundary conditions, the unstable wavenumbers are limited to the discrete values *k* = 2π*m*/*l* for *m* = 0, 1, 2…, where *m* is the mode or the number of aggregates [25,26]. As *l* corresponds to the length of the circumference of the vessel of radius *r*, *l* = 2π*r*, the wavenumber *k* can be expressed as the ratio of the number *m* of aggregates to the radius *r*, *k* = *m*/*r*.

If the stability matrix *A_k_* has at least one eigenvalue with a positive real part, then the homogeneous steady state is unstable. For the steady state to be unstable to spatial disturbances the determinant of the stability matrix has to be negative [25,50]:

A 2 $$\det(A_{k})=(Dk^{2}+\alpha )(k^{2}+\delta )-\chi k^{2}\gamma /{\left(1+\beta \right)}^{2}<0.$$

An analysis of det(*A_k_*) as a quadratic polynomial in the one variable *k*^2^ reveals the following necessary condition for the instability to occur:

A 3 $$\chi >\left(D\delta +\alpha +2\sqrt{D\delta \alpha }\right){\left(1+\beta \right)}^{2}/\gamma .$$

As one can see, this condition does not depend on the domain size, i.e. on the length *l* of the circumference of the vessel.

This instability condition can be used to obtain an interval (*k*_1_, *k*_2_) of unstable wavenumbers,

A 4 $$k_{1,2}^{2}=\kappa \pm \sqrt{\kappa^{2}-\delta \alpha /D},\;\text{where }\kappa =(\chi \gamma /{\left(1+\beta \right)}^{2}-D\delta -\alpha )/2D.$$

As *k* = *m*/*r,* where *m* is the integer, the number *m* of unstable aggregates varies from *m*_min_ to *m*_max_,

A 5 $$m\in [{m_{\min}}_{,}m_{\max}],m_{\mathrm{avg}}=(m_{\min}+m_{\max})/2,\;\text{where }m_{\min}=\text{ceil}(rk_{1}),m_{\max}=\text{floor}(rk_{2}),$$

where ceil and floor are the upward and downward rounding functions.

Taking into account equation (A 4), the average number *m*_avg_ of aggregates can be estimated as follows:

A 6 $$m_{\mathrm{avg}}\approx r\sqrt{\kappa }=r\sqrt{(\chi \gamma /{\left(1+\beta \right)}^{2}-D\delta -\alpha )/2D}.$$
